# Outcomes of COVID-19 in Patients With Lung Cancer Treated in a Tertiary Hospital in Madrid

**DOI:** 10.3389/fonc.2020.01777

**Published:** 2020-09-16

**Authors:** Antonio Calles, María Inmaculada Aparicio, Manuel Alva, Marianela Bringas, Natalia Gutierrez, Javier Soto, Marta Arregui, Victoria Clara Tirado, Enrique Luis Álvarez, María del Monte-Millán, Tatiana Massarrah, Mar Galera, Rosa Álvarez, Miguel Martín

**Affiliations:** ^1^Medical Oncology Department, Hospital General Universitario Gregorio Marañón, Madrid, Spain; ^2^Instituto de Investigación Sanitaria Gregorio Marañón, Madrid, Spain; ^3^CiberOnc, Madrid, Spain

**Keywords:** COVID-19, coronavirus, SARS-CoV-2, lung cancer, tocilizumab, multivariate, immunotherapy, targeted therapy

## Abstract

**Background:** Cancer patients represent a vulnerable population for COVID-19 illness. We aimed to analyze outcomes of lung cancer patients affected by COVID-19 in a tertiary hospital of a high-incidence region during the pandemic.

**Methods:** We annotated 23 lung cancer patients consecutively diagnosed with COVID-19 at our institution (HGUGM; Madrid, Spain) between March 4th, 2020 and May 12th, 2020. Only patients with a confirmatory SARS-CoV-2 RT-PCR were included in the study.

**Results:** All patients had at least 1 COVID-19 related symptom; cough (48%), shortness of breath (48%), fever (39%), and low-grade fever (30%) were the most common. Time from symptoms onset to first positive SARS-CoV-2 PCR was 5.5 days (range 1–17), with 13% of cases needed from a 2nd PCR to confirm diagnosis. There was a high variability on thoracic imaging findings, with multilobar pneumonia as the most commonly found pattern (74%). Main lab test abnormalities were low lymphocytes count (87%), high neutrophil to lymphocyte ratio -NLR- (78%), and elevated inflammatory markers: fibrinogen (91%), c-reactive protein -CRP- (87%), and D-dimer (70%). In our series, hospitalization rate was 74%, 39% of patients developed acute respiratory distress syndrome (ARDS), and the case-fatality rate was 35% (8/23). 87% of patients received anti-viral treatment (87% hydroxychloroquine, 74% lopinavir/ritonavir, 13% azithromycin), 43% corticosteroids, 26% interferon-β, 4% tocilizumab, and 82% of hospitalized patients received anticoagulation. High-oxygen requirements were needed in 39% of patients, but only 1 pt was admitted for invasive MV and was discharged 42 days after admission. Multiple variables related to tumor status, clinical baseline conditions, and inflammation markers were associated with mortality but did not remain statistically significant in a multivariate model. In patients with lung cancer receiving systemic therapy (*n* = 242) incidence and mortality from COVID-19 were 4.5, and 2.1%, respectively, with no differences found by type of treatment.

**Conclusions:** Lung cancer patients represent a vulnerable population for COVID-19, according to the high rate of hospitalization, onset of ARDS, and high mortality rate. Although larger series are needed, no differences in mortality were found by type of cancer treatment. Measures to minimize the risk of SARS-CoV-2 infection remain key to protect lung cancer patients.

## Introduction

In late 2019, severe acute respiratory syndrome coronavirus 2 (SARS-CoV-2) was newly reported in Wuhan, China and rapidly spread across the globe responsible for thousands of deaths in China, followed by other countries, like Italy and Spain. In March 11th, 2020, the World Health Organization (WHO) declared the new coronavirus disease 2019 (COVID-19) a pandemic.

First COVID-19 confirmed case in Madrid was detected on February 25th, 2020. Since then and until May 12th, 2020 there have been 65,269 confirmed cases by SARS-CoV-2 PCR, with 41,856 hospitalizations, 3,555 ICU admissions, and 8,760 reported deaths by COVID-19 in Madrid (1). A preliminary seroprevalence study revealed a 11.3% prevalence in Madrid by measuring IgG antibodies anti-SARS-CoV-2, accounting for one of the most prevalent regions in Spain (2). National lockdown in Spain took place on March, 15th 2020.

Even when most patients develop mild symptoms from COVID-19, or are asymptomatic, approximately 20% develop severe symptoms (3, 4). These severe clinical manifestations consist on acute respiratory distress syndrome (ARDS), septic shock, and multiorgan failure. In the pathogenic of severe COVID-19 cases underlies a cytokine release syndrome (CRS) in which interleukin 6 (IL-6) plays a central role (5, 6). In severe cases, admission in intensive care units (ICU) and mechanical ventilation (MV) is needed. No specific antiviral or anti-inflammatory therapy has demonstrated to date any clinical efficacy to reduce mortality of COVID-19. Most treatments remain experimental, and supportive care adapted to the clinical situation of the patient remains the basis of COVID-19 management. For the overall population, mortality for COVID-19 is estimated in 1–3% (4).

Cancer patients and cancer survivors represent a vulnerable population for COVID-19. Overall prevalence of COVID-19 in cancer patients is estimated on 1–6% and is higher than in non-cancer population (7–9). Furthermore, cancer patients have worse outcomes from COVID-19, supported by previous reports from China (7, 9, 10), Italy (11), and more recently New York (8). Risk of severe events defined as admission to the ICU, the need for MV or death is reported up to 39% of patients with cancer, and mortality rate could be as high as 30% (9). Moreover, age relates to COVID-19 lethality with a fatality rate of 15% for people 80 and over in European population (11). In particular, lung cancer patients seem to rank among the tumor type with highest risk of unfavorable outcomes for COVID-19. Reasons for these unfavorable outcomes in cancer patients with lung cancer are not fully elucidated, but as in other respiratory infections, cancer patients have an immune suppressed baseline condition, receive cancer therapies that can alter the immune response, and present with lung damage or lower lung capacity in relation to the disease itself or treatments received such as radiotherapy and surgery. In addition, lung cancer is an age-related disease and smoking is prevalent in patients with lung cancer. Lastly, patients with lung cancer have usually pulmonary and cardiovascular comorbidities. Altogether, patients with lung cancer accumulate all known factors associated to worse outcome from COVID-19 (12–15).

Differences in health-care systems, in the incidence and prevalence of SARS-CoV-2 infection by geographic regions, and patient access to intensive support care -including MV- and treatment with antivirals or anti-IL6/IL1 agents may ultimately influence outcomes in patients with lung cancer affected by COVID-19.

We aimed to describe the clinical characteristics of lung cancer patients with COVID-19 attended in a tertiary hospital in Madrid, one of the most hit regions by coronavirus in the world so far, and analyze factors associated with worse outcome, including type of treatment receiving at the time of COVID-19 diagnosis.

## Patients and Methods

This is an observational, retrospective, single-center study. We collected all lung cancer patients diagnosed with COVID-19 at Hospital General Universitario Gregorio Marañón, Madrid, Spain between February 24th, 2020 to May 12th, 2020. Patients must have a confirmatory SARS-CoV-2 reverse transcription polymerase chain reaction (RT-PCR) to be included in the study. Clinically suspected cases and cases with close contact to COVID-19 confirmed cases were not included if either not tested or tested negative by RT-PCR. Procedures for real time RT-PCR confirmation of SARS-CoV-2 infection have been described elsewhere (16).

Indications of SARS-CoV-2 RT-PCR followed institutional guidelines based on recommendations and case definition available at that moment by the Ministry of Health. Initially, this included only suspicious cases. Suspicious case was any person with a clinical presentation of acute respiratory infection of any severity that causes, among others, fever, cough, or of shortness of breath. Other symptoms such as odynophagia, anosmia, ageusia, muscle pain, diarrhea, chest pain, or headaches, among others, may also be symptoms of suspected SARS-CoV-2 infection according to clinical criteria. We performed SARS-CoV-2 RT-PCR to every suspicious case and included all lung cancer patients attended at our hospital (emergency room, hospitalization, ambulatory office, day care area). On March 19th, 2020 an entrance triage was stablished at the Medical Oncology Department consisting on both a questionnaire and temperature control. For either suspicious case or study of close contact, this triage visit was followed by SARS-CoV-2 PCR. In addition, every patient initiating any new cancer therapy was SARS-CoV-2 PCR tested before treatment administration but not subsequently tested if COVID-19 was not clinically suspected. Study of the contacts with confirmed COVID-19 persons corresponded to Public Health and Primary Care Physicians, with no access to this information initially under our registries if the patient was not referred to the hospital for clinical assessment.

For data collection, demographic and clinical features, pathology and molecular testing, most recent cancer therapy, laboratory and radiological data, treatment schemes, and outcomes were registered.

Patients were classified to be in active cancer treatment defined when <30 days from last dose of systemic therapy or <2 weeks from last dose of radiation therapy.

Routine blood examinations were measured at admission and included complete blood count, coagulation profile, and serum biochemistry (including liver function tests, creatine kinase -CK-, lactate dehydrogenase -LDH-, c-reactive protein -CRP-, and procalcitonin). We calculated the ratio of absolute neutrophil and lymphocyte counts (NLR) as a measure of systemic inflammatory stress (17). Temperature, and oxygen saturation by pulse oximeter (SpO2) were measured at admission. Fever was defined as 38°C or above whereas low-grade fever was defined as a temperature between 37°C and 37.9°C. IL-6 or IL-1β levels were not systematically tested, as these parameters were reserved only for patients before ICU admission.

Treatment for COVID-19 followed institutional approved protocols and is summarized in Supplementary Table 1. Protocols changed over time base on both current evidence and drug availability. For patients requiring hospitalization, combination of hydroxychloroquine plus lopinavir/ritonavir was considered as institutional standard of care. Association with intravenous antibiotic was recommended to avoid bacterial superinfection (ceftriaxone was the antibiotic of choice unless other risk factors were present). Until drug shortage on March 24th, interferon β-1b was also administered (8 x 10^6^ IU subcutaneously every 48 h up to 3 doses). Methylprednisolone or dexamethasone was administered only in cases of both clinical and radiological worsening. Tocilizumab was only indicated in candidates for either invasive (IMV) or non-invasive ventilation together with either interleukin-6 levels >40 pg/ml o D-dimer >1,500 U/ml. Remdesivir was only indicated for patients requiring IMV. Thromboprophylaxis was not mandatory initially by institutional guidelines and followed general recommendations for cancer patients. For ambulatory patients, either hydroxychloroquine plus lopinavir/ritonavir or hydroxychloroquine plus azithromycin was recommended.

Outcomes measured included hospitalization rate, maximum oxygen requirement, organ failure, ICU admission, use of mechanical ventilation, and death of any cause.

After symptoms recovery, patients were tested weekly by RT-PCR before resuming any cancer therapy or visit to the outpatient office due to an entrance triage stablished at the Medical Oncology Department.

### Statistical Analysis

Associations between categorical data were tested using Chi-square or Fisher's exact test; Wilcoxon rank sum test or unpaired *t* test were used for calculations for association with continuous measures. All tests were conducted at the two-sided 0.05-level with no adjustments for multiple comparisons. We assessed all study variables to analyze correlations with mortality using logistic regression. Correlation between variables was assessed by the Kendall rank correlation method. Correlation plot was drawn with corrplot package. Median confidence intervals were calculated by bootstrapping method with function MedianCI from DescTools package. We conducted receiver-operator characteristic (ROC) curve analysis for NLR. An optimal cut-off to differentiate high-risk versus low-risk groups was determined using the Youden method; sensitivity, specificity, and Youden's index were also reported. All analyses were performed using Prism 8 (version 8.4.3) and R (version 4.0.1).

This study was approved by the local Ethics Committee with registration number GOM-HGUGM-2020-04.

## Results

### Demographic and Clinical Characteristic of Lung Cancer Patients With COVID-19

**Figure 1:**
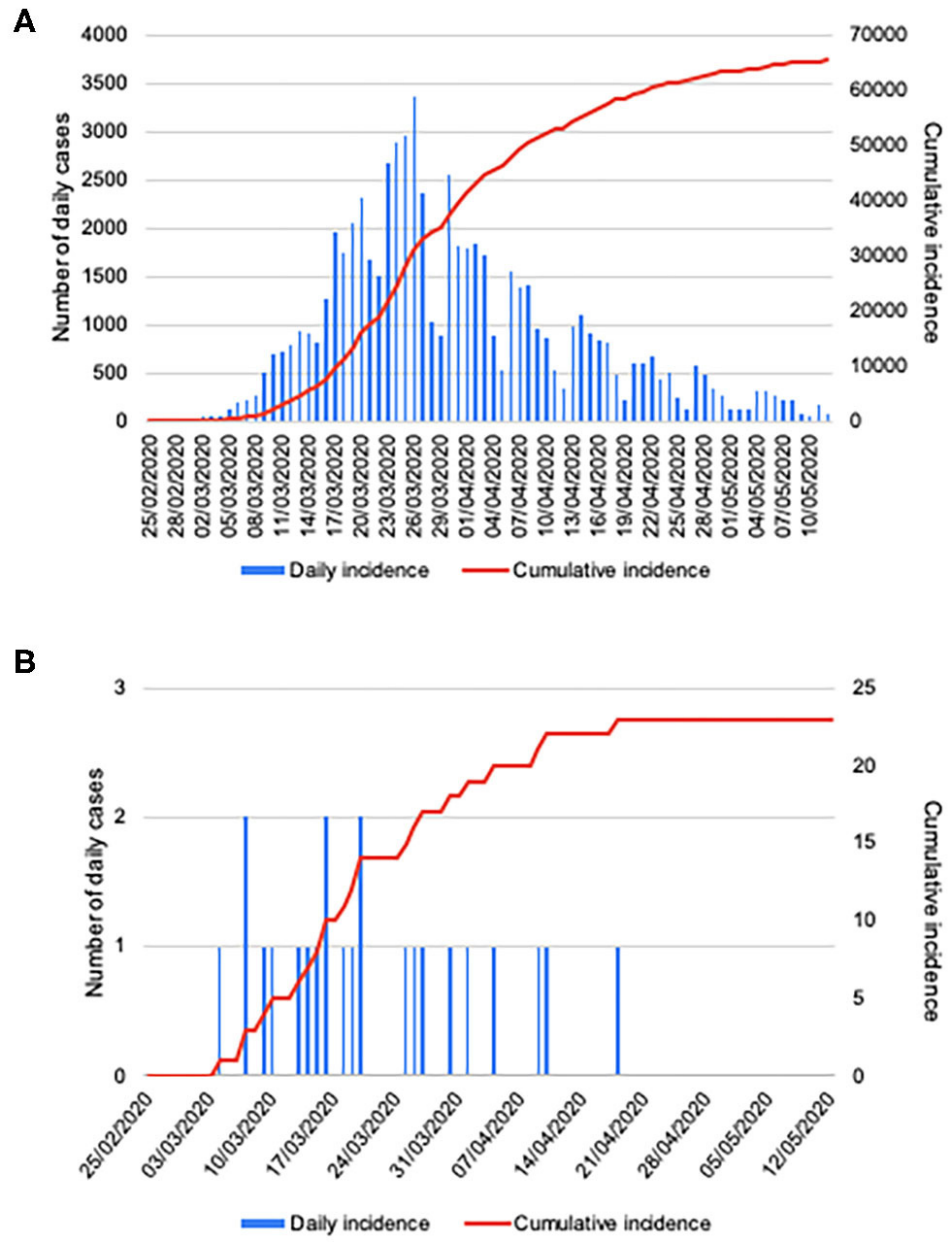
Confirmed COVID-19 cases by PCR. Daily new cases, and cumulative incidence in Madrid* **(A)** and in patients with lung cancer attended at Hospital General Universitario Gregorio Marañón **(B)**, from February 25th, 2020 to May 12th, 2020. *Source: General Directorate of Public Health (1).

First SARS-CoV-19 confirmed lung cancer patient in our institution was on March 5th, 2020. Since then a total of 23 more cases were detected as per May 12th, 2020. Daily and cumulative incidence of COVID-19 in lung cancer patients at our institution is comparable to epidemiologic data in Madrid during these 10 weeks (Figure 1) (1). Of note, all lung cancer patients included in our series corresponded to suspicious cases and no lung cancer patient was diagnosed of COVID-19 from the upfront triage stablished at the entrance of the Medical Oncology Department.

Clinical features of the 23 COVID-19 lung cancer patients resemble average demographics for lung cancer patients (Table 1). Median age was 66 years-old (range 49–86), predominantly male (78%), and past smoking history (87%). Up 87% of patients had at least 1 comorbidity (range 1–7). Most common comorbidities were hypertension (57%), diabetes (39%), and chronic obstructive pulmonary disease—COPD—(30%). Cardiac dysfunction, chronic renal disease, and chronic liver disease were present in 26, 17, and 17%, respectively (Table 1). Two patients (9%) had past tuberculosis infection. Up to 22% of patients had immunosuppressive conditions: 1 patient with HIV infection, 1 patient with liver transplantation under immunosuppressants, 3 patients on chronic steroid intake >10 mg prednisone per day. Only 3 patients were on permanent anticoagulants at the time of COVID-19 diagnosis.

**Table 1:** Demographic, pathology, and treatment characteristics of lung cancer patients with COVID-19 (*n* = 23).

	**No**.	**%**
Age (years)		
Median (range)		66 (49-86)
40-49	1	4%
50-59	3	13%
60-69	12	52%
70-79	3	13%
80-89	4	17%
Gender		
Male	18	78%
Female	5	22%
Body Mass Index (BMI)		
Mean (range)		25.65 (19.57-33.96)
Underweight (<18.5)	0	0%
Normal (18.5-24.9)	10	43%
Overweight (25.0-29.9)	8	35%
Obese class I (30.0-34.9)	5	22%
Obese class II (35.0-39.9)	0	0%
Obese class III (≥40.0)	0	0%
Smoking Status		
Current	8	35%
Former	12	52%
Never	2	9%
Unknown	1	4%
ECOG PS		
0	10	43%
1	8	35%
2	5	22%
>2	0	0%
Comorbidities		
Median (range)		3 (0-7)
No comorbidities	3	13%
Hypertension	13	57%
Diabetes	9	39%
COPD	7	30%
Cardiac dysfunction^a^	6	26%
Chronic renal disease	4	17%
Chronic liver disease	4	17%
Dyslipidemia	3	13%
Coronary heart disease	2	9%
Peripheral vascular disease	2	9%
Cerebrovascular disease	1	4%
Others^b^	11	48%
Immunosupression^c^	5	22%
Past TB infection/vaccine	2	9%
Chronic anticoagulation therapy	3	13%
Pathology		
Adenocarcinoma	14	61%
SqCC	6	26%
SCLC	2	9%
Others (LCNEC)	1	4%
Molecular profiling		
EGFR	1	4%
ALK	1	4%
ROS1	1	4%
Triple wild-type	9	39%
Other (BRAF)	1	4%
Not available^d^	10	43%
PD-L1 status		
<1%	6	26%
1-49%	5	22%
≥50%	5	22%
Unknown^e^	7	30%
Stage		
I-II	1	4%
III	8	35%
IVA	5	22%
IVB	9	39%
Metastasis		
# metastasic sites		
Median (range)		1 (1-4)
Location		
Lung/Pleural	12	52%
Liver	3	13%
Brain	1	4%
Other visceral	3	13%
Bone	6	26%
Lymph nodes	8	35%
Other^f^	2	9%
Treatment		
Systemic^1^	11	48%
Chemotherapy	4	17%
Targeted therapy	1	4%
Immunotherapy	5	22%
Chemo+Immunotherapy	0	0%
Chemoradiation	1	4%
Radiation^2^	7	30%
Lung	1	4%
Brain (WBRT/PCI)	2	9%
Bone (palliative)	3	13%
Other (prostate)	1	4%
No systemic treatment	12	52%
Pending of treatment initiation^g^	6	26%
Drug holidays	2	9%
Surveillance	4	17%

a Atrial fibrillation, aortic stenosis, hypertrophic cardiomyopathy;

b Liver transplantation, HIV, previous TB infection, BPH, prostate cancer, hypothiroidism, pulmonary embolism, reumathoid arthritis;

c HIV, chronic steroid intake or immunosupresants;

d SqCC, 5; SCLC, 2; non-SqCC, 2; LCNEC, 1;

e includes 3 non-NSCLC/2 SCLC + 1 LCNEC;

f includes adrenal, pericardium, kidney, extra thoracic lymph nodes;

g *5 patients recently diagnosed with lung cancer waiting for 1^st^ line; 1 pt SCLC relapse for 2^nd^ line*.

1 Last dose in 30 days before the onset of symptoms;

2 *Up to 2 weeks before symptoms initiation*.

*PS, performance status; COPD, chronic obstructive pulmonary disease; TB, tuberculosis; SqCC, squamous cell carcinoma; SCLC, small cell lung cancer; LCNEC, large cell neuroendocrine carcinoma; EGFR, epidermal growth factor receptor; ALK, anaplastic lymphoma kinase; WBRT, whole brain radiation therapy; PCI, prophylactic cranial radiation*.

Most common histology was adenocarcinoma (61%), squamous carcinoma (26%), and small-cell lung cancer (9%) (Table 1). In non-squamous non-small-cell lung cancer, 9/15 were all EGFR, ALK, and ROS1 wild-type. One case of each EGFR, ALK, ROS1, and BRAF genetic alteration were found in the COVID-19 patients. In tumor specimens with available PD-L1 tumor expression, COVID-19 cases were equally distributed in <1%, 1-49%, and ≥50% subgroups.

Vast majority of patients were locally advanced or metastatic stage at the time of COVID-19 illness (Table 1). Sixty-one percent of patients were stage IV (22% stage IVA, 39% stage IVB), whereas 35% were stage III. Median number of metastatic sites was 1 (range 1–4). Most common sites of metastasis were present at lung/pleural (52%), lymph nodes (35%), bone (26%), and liver (13%).

Seventy percent of patients were on either systemic therapy or radiation therapy at the time of COVID-19 diagnosis (Table 1). Types of therapy were systemic therapy alone in 10/23 patients (44%), concurrent radical chest chemoradiation in 1 (4%), and non-thoracic radiation therapy in 6/23 patients (26%). Nearly half of the patients were receiving systemic anti-cancer treatment. Most common systemic therapies were chemotherapy in 5/23 (22%; includes 1 patient on concurrent thoracic chemoradiation), immunotherapy in 5/23 (22%), and targeted therapy in 1/23 (4%). No patients with COVID-19 were found to be on combined chemo-immunotherapy in our series. Non-thoracic radiation therapy was delivered with the following indications: palliative for bone metastasis (*n* = 3), brain (*n* = 2, 1 PCI, 1 palliative WBRT), and prostatic (*n* = 1) for localized prostate cancer radical therapy.

Of the 12 patients not receiving any systemic anti-cancer treatment, 5 patients recently diagnosed of lung cancer were pending on first-line treatment initiation. Moreover, 1 patient with recently confirmed relapse of small-cell lung cancer (SCLC) was pending on second-line chemotherapy treatment. Additionally, 2 patients with metastatic disease were in a “drug-holiday” period. Finally, 4 patients were under surveillance with no evidence of disease. Overall, most lung cancer patients had an active tumor at the time of COVID-19 diagnosis, and only 4 patients (17.4%) were considered to be without active tumor present.

### Clinical Presentation of COVID-19 in Lung Cancer Patients

All lung cancer patients had at least 1 COVID-19 related symptom (median 3; range 1–4); cough (48%), shortness of breath (48%), fever (39%), and low-grade fever (30%) were the most commonly reported COVID-19 symptoms (Table 2). Time from symptoms onset to first positive SARS-CoV-2 PCR was 5.5 days (range 1–17). Most cases were confirmed at first SARS-CoV-2 PCR, however in 2 cases (13%) a second PCR was required to confirm diagnosis.

**Table 2:** Clinical Presentation of COVID-19 in patients with lung cancer (*n* = 23).

			**No**.		**%**
Symptoms at diagnosis					
Cough			11		48%
Shortness of breath			11		48%
Fever (≥38°C)			9		39%
Low grade fever (37°-37.9°C)			7		30%
Productive cough			5		22%
Fatigue			5		22%
Myalgia			5		22%
Headache			2		9%
Sore throat			1		4%
Diarrhea			1		4%
Anosmia			1		4%
Disgeusia			1		4%
Asymptomatic			0		0%
Time from symptoms onset to positive SARS-CoV-2 PCR					
5.5 days (range 1-17)					
Number of PCRs needed to COVID-19 confirmation					
			1		87%
			2		13%
Temperature at diagnosis					
Median (range)			37.8°C		(36.0-39.0°C)
Oxygen saturation at diagnosis^a^					
Median (range)			92.5%		(65%-98%)
>95%			8		35%
90-95			6		26%
<90			8		35%
**Lab test at diagnosis**^a^	**Units**	**Median**	**Lower 95% CI**	**Upper 95% CI**	**Normal range**
Hemoglobin	g/dl	12.6	11.2	13.8	13.0-17.5
Leucocytes	x10^3^/μl	6.07	4.0	8.0	4.00-10.00
Neutrophils	x10^3^/μl	4.6	2.7	6.2	1.8-7.5
Lymphocytes	x10^3^/μl	0.7	0.3	0.9	1.3-3.5
Platelets	x10^3^/μl	215.5	140	302	140-400
Protrombin time	sec	12.6	11.8	14.2	10.5-13.5
Fibrinogen	mg/dL	624	563	739	150-450
D-Dimer	ng/ml	551	303	830	0-250
Lactate	mmol/L	1.9	1.57	3.8	0.6-2.2
Sodium	mmol/L	136	132	138	135-145
ALT	U/L	18	12	29	5-41
CK	U/L	37	23	72	39-308
Ferritin	μg/L	1103	327	2223	22-274
CRP	mg/dL	7.25	2.8	17	0.0-0.5
Procalcitonin	μg/L	0.07	0.05	0.29	0.00-0.50
NLR		6.94	3.38	13.75	1-3
**Thoracic Imaging**^a^			**No**.		**%**
Chest X-ray			19		83%
No pneumonia			4		17%
Unilobar pneumonia			1		4%
Multilobar pneumonia			14		61%
CT scan^*^			3		13%
Multilobar pneumonia			3		13%

a *n = 22; 1pt had no lab test and chest x-ray performed*.

* *PET-CT scan in 1 case*.

*ALT, alanine transaminase; CK, creatine kinase; CRP, c-reactive protein; NLR, neutrophil to lymphocyte ratio; CT, computed tomography*.

Temperature at diagnosis was above 38°C in 8 patients (35%), between 37.0°C and 38°C in 9 patients (39%), and below 37°C in 6 patients (26%). Oxygen saturation (SpO2) at the time of diagnosis was above 95% in 8 patients (35%), between 90 and 95% in 6 patients (26%), and below 90% in 8 patients (35%). Main lab test abnormalities found were low lymphocytes count (87%), low hemoglobin, high NLR (78%), and elevated inflammatory markers: fibrinogen (91%), D-dimer (70%), CRP (87%), and ferritin (Table 2). To further evaluate the predictive value of NLR based on a dichotomous classification, we conducted ROC curve analysis. With an area under the curve of 0.804, the value that maximized the Youden's index was 10.690. The specificity and sensitivity for this value was 0.857 and 0.750, respectively (Supplementary Figure 1).

**Figure 2:**
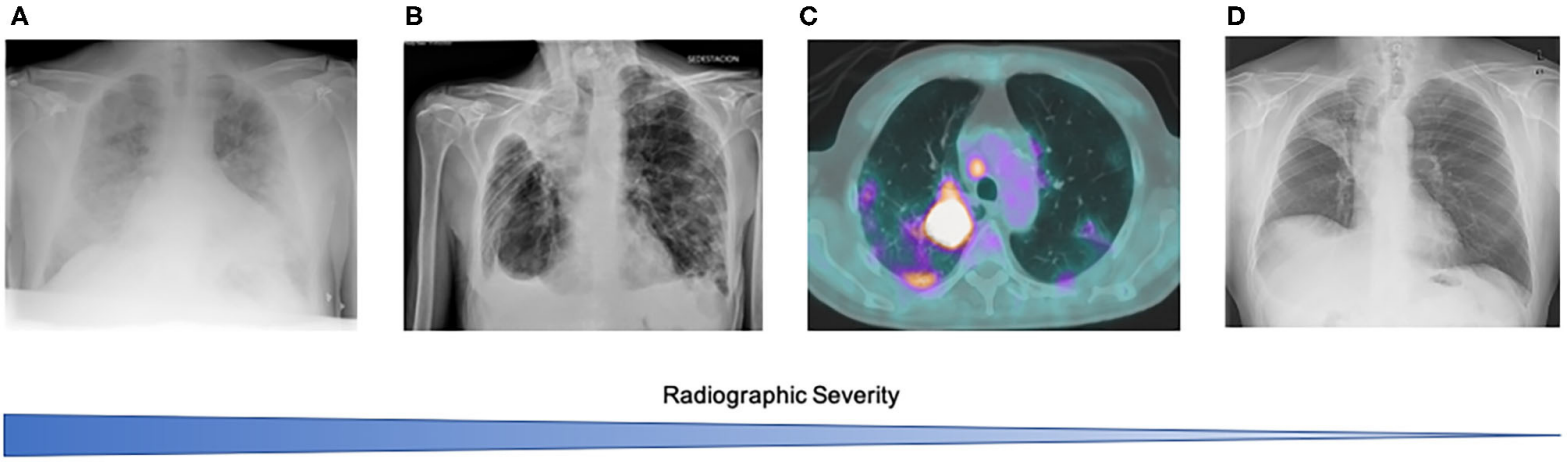
Radiological findings at the time of diagnosis of COVID-19 in patients with lung cancer. **(A)** Severe bilateral pneumonia in a stage IV ALK rearranged lung adenocarcinoma patient on first line alectinib; **(B)** Bilateral pneumonia in an 84 years-old male, stage IV lung adenocarcinoma in second line treatment with pembrolizumab. Residual findings of previous right empyema; **(C)** Incidental finding in a PET-CT scan performed as part of radiotherapy treatment planning in a stage IIIB locally advanced NSCLC; **(D)** No evidence of infiltrates in a stage IV lung adenocarcinoma patient on second line pembrolizumab. Patient was on previous antiretrovirals due to HIV. Obstructive upper right lobe atelectasis. Outcome was full COVID-19 recovery for patients in **(B–D)** and they could start/resume their anti-cancer treatment. Patient in **(A)** died from COVID-19 ARDS and massive GI bleeding.

There was a high variability on thoracic imaging findings at diagnosis (Figure 2, Table 2). Most cases had only a chest X-ray as initial radiologic assessment (*n* = 19). Only one case did not have any radiologic imaging performed. In the 3 cases where a computed tomography (CT) scan was available, COVID-19 pneumonia was found as an incidental finding, one of them in a PET-CT scan used for radiotherapy treatment planning in a locally advanced non-small cell lung cancer (NSCLC). In chest X-ray, findings were as follow: no pneumonia (*n* = 4), unilobar pneumonia (*n* = 4), multilobar bilateral pneumonia (*n* = 14). All 3 patients with chest CT-scan had multilobar pneumonia. Overall, multilobar pneumonia was the most common radiology pattern found in 74% of cases.

### Outcomes of Lung Cancer Patients With COVID-19

**Figure 3:**
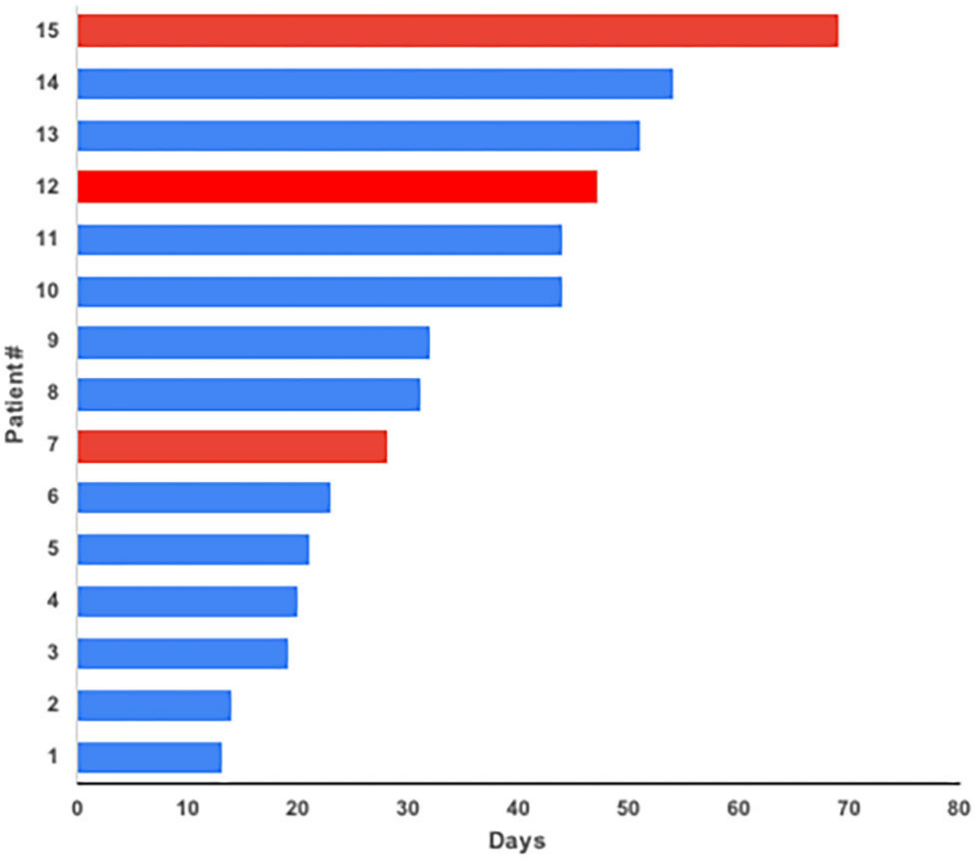
Time to negative SARS-CoV-2 PCR from 1st symptom of COVID-19. On COVID-19 survivor patients, median time to negativizing SARS-CoV-2 PCR from 1st COVID-19 symptom was 31 days (range 13–69). There were 3 cases remaining PCR positive at the time of the database lock (in red).

In our series, 17/23 patients required admission which represents a hospitalization rate of 74%. Median hospitalization stay was 16 days (range 1–44). To database lock date, 12 patients are considered cured (52%), 8 patients have died (35%), and 3 cases remain in 30 days follow-up period (13%). None of the 6 patients managed in ambulatory basis have died. On COVID-19 survivor patients, median time to negativizing SARS-CoV-2 PCR from 1st COVID-19 symptom was 31 days (range 13–69). There were 3 cases remaining PCR positive at the time of the database lock (Figure 3).

Overall, 20/23 patients (87%) received anti-viral treatment (Table 3): 20 hydroxychloroquine (87%), 17 lopinavir/ritonavir (74%), 3 azithromycin (13%). In ambulatory patients, hydroxychloroquine plus lopinavir/ritonavir (*n* = 2), hydroxychloroquine + azithromycin (*n* = 1), or no specific treatment (*n* = 3) were used. Immunomodulators were used as follow: 6 patients received IFN-β (26%), 10 corticosteroids (43%), and 1 tocilizumab (4%). The only patient receiving tocilizumab was on cancer surveillance with no evidence of disease after definitive chemorradiation completion 6 months before, and was receiving local radiation therapy for local prostate cancer at the time of COVID-19 diagnosis. Most used antibiotics were ceftriaxone (*n* = 10), meropenem (*n* = 1), piperacilin/tazobactam (*n* = 1).

**Table 3:** Outcomes of lung cancer patients with COVID-19 (*n* = 23).

	**No**.	**%**
Hospital admission	17	74%
Evolution		
Cured	12	52%
Died	8	35%
Ongoing	3	13%
Anti-viral treatment	20	87%
LPV/r + HCLQ	17	74%
With antibiotics^*^	9	39%
With IFN-β + ceftriaxone	3	13%
HCLQ + azithromycin	2	9%
HCLQ only	1	4%
Tocilizumab	1	4%
Corticosteroids	10	43%
Anticoagulants	14	61%
Maximum oxygen requirements		
None	6	26%
Low-flow	8	35%
Ventimask	7	30%
Optiflow	1	4%
Mechanical ventilation	1	4%
Organ failure		
None	14	61%
ARDS	9	39%
Shock	1	4%
Renal	1	4%
Neurologic	1	4%
Time to ARDS (from symtomps onset)		
6 days (range 1-16)		

* *Ceftriaxone (n = 6), ceftriaxone + azithromycin (n = 1), meropenem (n = 1), or piperacilin/tazobactam (n = 1)*.

*LPV/r, lopinavir/ritonavir; HCLQ, hydroxychloroquine; IFN-β, Interferon beta 1b; ARDS, acute respiratory distress syndrome*.

Maximum oxygen requirements were none in 6 patients (26%, all ambulatory), low-flow (nasal cannula) in 8 patients (35%), large-reservoir Venturi masks to 15 l/min (Ventimask) in 7 patients (30%), high-flow (Optiflow™) in 1 pt (4%), and IVM in 1 pt (4%).

Nine patients (39%) developed organ failure during COVID-19 course. All 9 cases included ARDS (39%), with 1 patient developing additional shock and renal impairment, 1 patient had a massive upper GI tract bleeding, and another patient developed neurologic disfunction. Median time to ARDS initiation was 6 days from COVID-19 symptoms onset (range 1–16), which is in line with the known time to expected cytokine storm described for severe COVID-19 patients. Only 1 patient was admitted in the ICU for MV and received tocilizumab and was discharged from the hospital 42 days later. In 14/17 hospitalized patients (82%) anticoagulation with enoxaparin was prescribed. No thromboembolic events were diagnosed during hospitalization.

### Predictors of Mortality in Lung Cancer Patients With COVID-19

**Figure 4:**
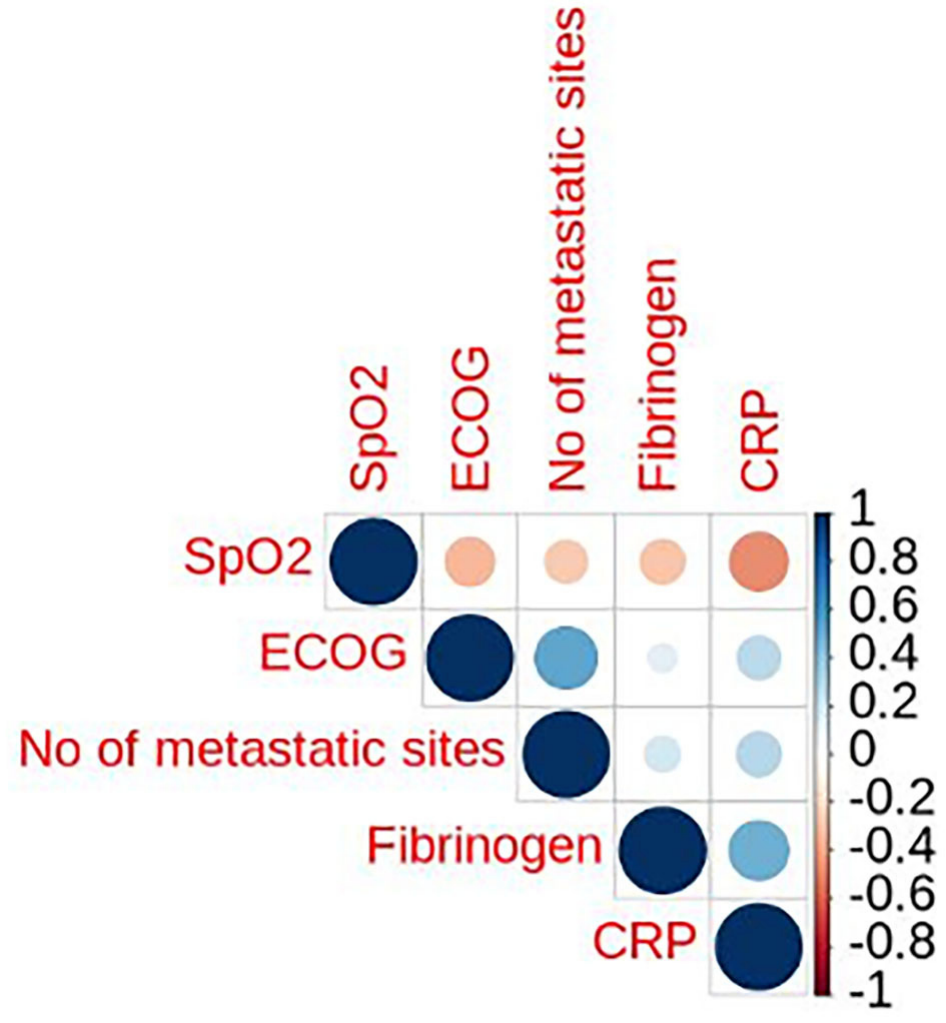
Map of correlations among variables associated with the outcome of death by COVID-19 in patients with lung cancer. Positive correlations are displayed in blue and negative correlations in red color. Color intensity and the size of the circle are proportional to the correlation coefficients. Correlation coefficient is indicated in the scale on the right. ECOG, Eastern Cooperative Oncology Group scale of performance status; SpO2, oxygen saturation at admission; CRP, c-reactive protein.

The case fatality rate (CRF) in our series was 35% (8/23 patients), and was higher in patients actively receiving anti-cancer treatment compared to those that not (5/11 patients, 45% vs. 3/12 patients, 25%, respectively; *p* = 0.4003). We aimed to analyze predictive factors of mortality based on baseline clinic-pathologic features, COVID-19 clinical presentation, and cancer treatment. Univariate analysis demonstrated a statistically significant association with mortality for oxygen saturation at diagnosis, ECOG performance status, number of comorbidities, stage IV, number of metastatic sites, progressive disease, and elevated CRP and fibrinogen (Table 4). However, there was strong correlation among multiple variables (Figure 4) and none of the predictive factors identified in the univariate analysis remained statistically significant in a multivariate model.

**Table 4:** Predictive factors of mortality for COVID-19 in patients with lung cancer by logistic regression (*n* = 23).

	**Univariate**	
**Variable**	**OR**	***P* value**
ECOG ≥1	10.50	0.049
SpO2	1.30	0.021
No. of metastasic sites	2.84	0.019
Fibrinogen	1.01	0.009
CRP	1.20	0.016
Progressive tumor^*^	∞	0.019
Stage IV^*^	∞	0.007

*OR, odds ratio; SpO2, oxygen saturation at admission; CRP, c-reactive protein*.

* *Values for these variables obtained with Fisher's exact test*.

**Figure 5:**
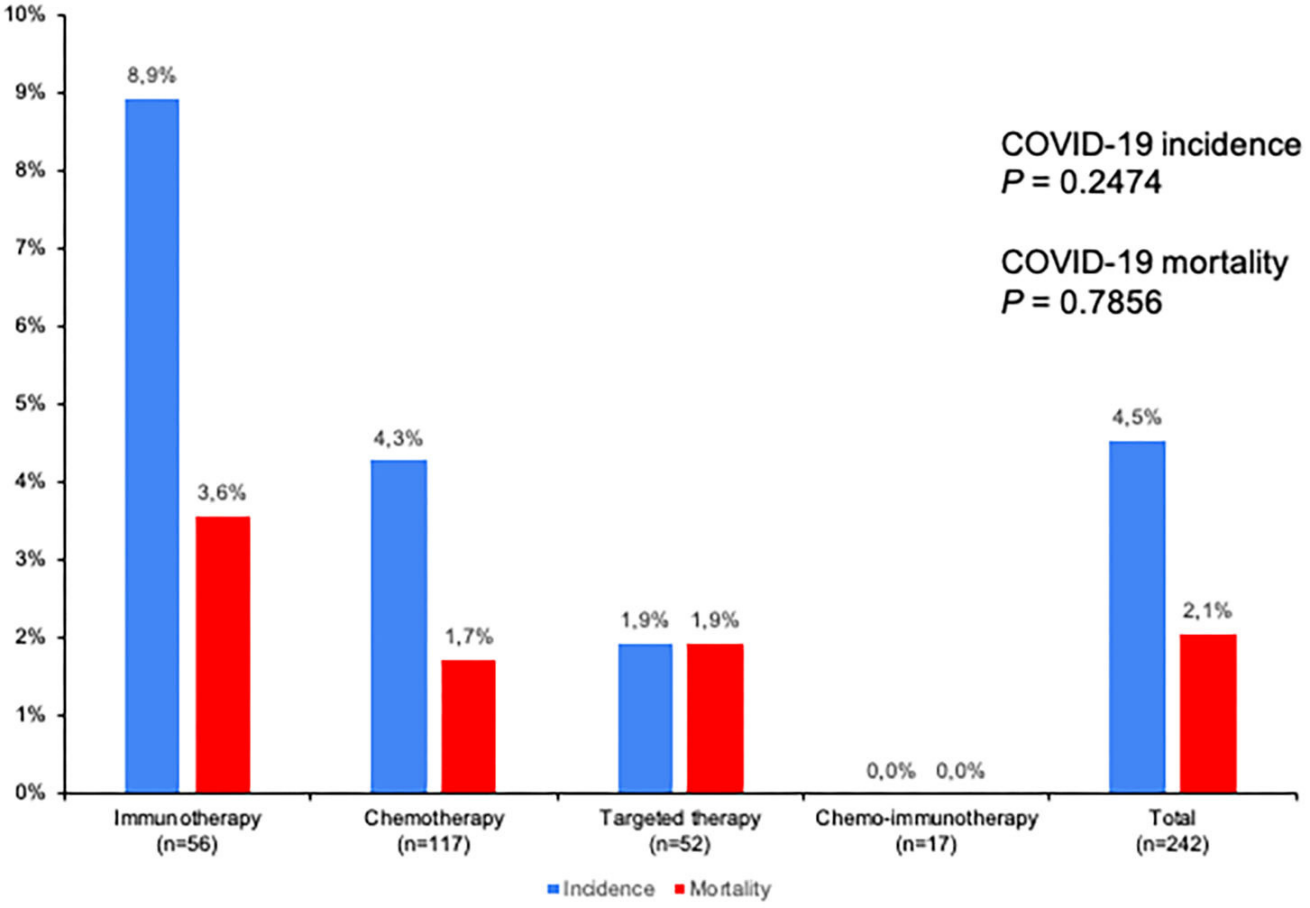
COVID-19 incidence and mortality by treatment type. 242 patients with lung cancer were receiving systemic anti-cancer therapy during COVID-19 outbreak from February to May 2020 in HGUGM, Madrid (Spain). Type of systemic treatment received was chemotherapy in 117 patients (48%), immunotherapy in 56 patients (23%), targeted therapy in 52 patients (22%), and chemo-immunotherapy in 17 patients (7%). Overall estimated incidence of COVID-19 was 4.5% and mortality 2.1%. No statistically differences were found by type of treatment for either COVID-19 incidence (*p* = 0.2474) or mortality (*p* = 0.7856).

We next tried to estimate both the incidence and mortality from COVID-19 in lung cancer patients that were in active treatment. We accessed to all the treatment records of lung cancer patients in our institution during the period of time of the outbreak. We found a total of 242 patients that were actively receiving systemic therapy. Men on cancer treatment were more frequently infected by COVID-19 than women (91 vs. 9.1%; OR 8.33, *p* = 0.047). There were no other differences in clinic-demographic characteristics among those lung cancer patients on cancer treatment with COVID-19 or not (Supplementary Table 2). Type of systemic treatment received was chemotherapy in 117 patients (48%), immunotherapy in 56 patients (23%), targeted therapy in 52 patients (22%), and chemo-immunotherapy in 17 patients (7%); 28 patients were on definitive thoracic concomitant chemoradiation (including 4 patients in radiation-immuno-chemotherapy). COVID-19 incidence and mortality were 4.5 and 2.1%, respectively, among all lung cancer patients that were actively receiving systemic therapy. Although patients receiving immune-checkpoint inhibitors had numerically higher both COVID-19 incidence and mortality (8.9 and 3.6%, respectively), no statistically significant differences were observed in either COVID-19 incidence or mortality by type of treatment (*p* = 0.2474 and *p* = 0.7856, respectively; Figure 5).

## Discussion

We described the clinical characteristics and outcomes on 23 lung cancer patients with COVID-19 attended in a tertiary hospital in Madrid during the pandemic. Clinical characteristics in our series of lung cancer patients with COVID-19 did not differ from what is expected in a Medical Oncology Service in a tertiary hospital. Most patients had comorbidities, were stage IV disease, predominantly male, had smoking history, and were on active cancer treatment. All patients had at least 1 COVID-19 related symptom, but not all of them developed pneumonia. These could be partially explained at least because not all patients had a thoracic CT scan at diagnosis which is more sensitive than chest X-ray to find lung abnormalities caused by SARS-CoV-2 infection. Main lab test abnormalities found in the majority of patients were low lymphocytes count, high NLR, and elevated markers of systemic immflamation: fibrinogen, ferritin, CRP, and D-dimer. These variables have been described as immunological markers correlated with COVID-19 severity (18). In contrast, we found procalcitonin, CPK, prothrombin time and other blood cell series than lymphocytes were not affected during SARS-CoV-2 infection in patients with lung cancer. At this point, we lack of enough information on IL-6 and IL-1β levels and CD4/CD8 cells counts, as only a minority of patients get these variables analyzed as were not consistently tested for all patients in our institution.

Patients with lung cancer were extremely vulnerable to COVID-19 based on a 74% hospitalization rate, 39% of patients developed ARDS, and a 35% case-fatality rate. In patients with lung cancer receiving active cancer treatment, COVID-19 had a 4.5% incidence and 2.1% mortality, and COVID-19 was most frequent in male (OR 8.3). Type of cancer treatment did not seem to correlate with COVID-19 outcomes. Hydroxychloroquine and lopinavir/ritonavir were the most frequently used drugs to treat COVID-19 in our patients. In addition, short courses of steroids were used in severe COVID-19 cases. We did not find any association with outcomes based on the therapy received to treat COVID-19, although we cannot address formal conclusions by the limited sample size of our series. Most of hospitalized patients in our series were anticoagulated at admission, which may account for the absence of thromboembolic events observed. However, we did not perform any autopsies to rule out the presence of unexpected thrombosis. Most recent evidence relates COVID-19 with higher incidence of thrombosis due to the interplay with inflammation and endothelial cells provoked by the SARS-CoV-2 (19–21), and suggests a rational use of prophylactic anticoagulants (22).

Madrid has a preliminary SARS-CoV-2 seroprevalence of 11.3%, it is the region with the highest mortality by COVID-19 in Spain and is among the most hit regions by the pandemic in the world to date (1). This epidemic situation stressed the health-care system to collapse. In our institution more than 1,100 out of the 1,350 beds were COVID-19 dedicated beds, expanded over 100 ICU beds, and more than 4,500 COVID-19 patients were admitted during the period of this study. In this triage situation, with limited ICU beds and ventilators, patients with previous life-limiting medical conditions were not usually admitted for intensive care. Our first lung cancer SARS-CoV-2 PCR confirmed case was detected only 9 days after the 1st officially confirmed case in Madrid. Evolution of the incidence in our hospital was in parallel to Madrid region. We reached the peak of incidence in the 2nd week of March, achieving 83% of cumulative cases with lung cancer and COVID-19 in our hospital 4 weeks after the start of the outbreak. Despite of this situation, continuity of care for patients with lung cancer at the Medical Oncology Department was maintained and the estimated incidence and mortality by COVID-19 in patients receiving active treatment was 4.5 and 2.1% which was below the average compared to the community. However, our COVID-19 diagnosis was based on SARS-CoV-2 RT-PCR in nasopharyngeal swabs and may underestimate the real prevalence of COVID-19 in lung cancer patients in our area. In this regard, an ongoing study promoted by the Spanish Lung Cancer Group (SLCG) is evaluating SARS-CoV-2 IgG seroprevalence focused on the lung cancer population (GRAVID study) and will provide more accurate information on real prevalence in this group of patients in Spain.

Initial reports from China indicated that patients with lung cancer had worse outcomes from COVID-19 (7, 9, 23), but limited information was available from other regions on the world. More recently, evidence is added to the previously reported from China. In New York city, Memorial Sloan Kettering Cancer Center (MSKCC) identified 102 consecutive lung cancer patients diagnosed with COVID-19 confirmed with SARS-CoV-2 PCR (24). Median age was 68 (range 31–91), 52% were female, 64% smoking history, and 72% had active or metastatic lung cancer. Low absolute lymphocyte count was the most remarkable finding in the lab test at baseline. 34% of patients received hydroxychloroquine for COVID-19 treatment, 62% of patients required hospitalization and 25% died from COVID-19. Overall, these cases represented 11% of all deaths among patients with lung cancers at their institution. Similarly, our rate of hospitalization and case-fatality rate fall in range to recently communicated preliminary data in TERAVOLT (25). This is a global registry of patients with thoracic malignancies affected by COVID-19 that involved a total of 200 cases from 21 countries around the globe. This study included not only SARS-CoV-2 PCR confirmed cases but also suspected COVID-19 cases, which accounted for 9% of the total. Again, characteristics of patients are similar to our series, with predominantly male (70.5%), current/former smoker (81.1%), stage IV (73.5%), NSCLC histology (75.5%), and 83.8% presented with comorbidities. 73.9% of patients were on active oncologic treatment (TKI 19%, chemotherapy 32.7%, ICI 23.1%, chemo-immunotherapy 13.6%). Most patients presented with fever, cough, and dyspnea, and 79.6% had pneumonia as in our series. COVID-19 in lung cancer patients lead to hospitalization in 76% of the cases, ARDS was reported in 26.8% of patients, and death occurred in 34.6%. Only 8.8% of patients were admitted in the ICU, and only 4.5% received IVM. Overall, our results are in line with others' experience, and confirms that lung cancer confers high-risk of worse outcomes of COVID-19. More information will soon become available from SOLID study, a national-wide registry involving more than 400 COVID-19 infected patients with thoracic malignancies in Spain.

Despite of high risk of complications by COVID-19 in patients with lung cancer, there were 6 patients who were successfully managed in ambulatory basis with mild evolution and not requiring hospitalization. Three cases were receiving treatment for advanced disease (2 with single agent immune checkpoint inhibitor, 1 with pemetrexed), two patients had active tumor without treatment (1 newly diagnosed stage III NSCLC, 1 mediastinal relapsed SCLC), and one case was under cancer surveillance without evidence of disease. Interestingly, two cases with past treated tuberculosis infection did not required hospitalization and survived COVID-19 without complications. Natural infection has been proposed to have a more tolerant immune system avoiding clinical worsening (26, 27). In addition, one HIV infected patient on chronic antiretrovirals had mild symptomatic COVID-19 disease, again successfully outpatient managed. HIV infection has not been reported as a condition for higher mortality for COVID-19, and antiretroviral therapy has been proposed as a protective factor for SARS-CoV-2 infection (28). In contrast, 2 out 5 patients with newly diagnosed lung cancer pending on systemic treatment initiation died due to COVID-19. The presence of an active cancer and the need for multiple procedures and hospital visits during the period of diagnosing a patient with lung cancer stresses the concept that minimizing visits and exposure may be critical to protect patients from SARS-CoV-2 infection.

The risk of severe COVID-19 in association with cancer treatments has conflictive results. We detected a higher incidence and mortality from COVID-19 in patients receiving immunotherapy with immune-checkpoint inhibitors (8.9 and 3.6%, respectively), although no statistically significant differences were observed by type of treatment (chemotherapy, targeted therapy, chemo-immunotherapy). Data from Wuhan, in China, showed that active cancer treatment received in the 14 days before SARS-CoV-2 infection had an increase on the risk of severe outcomes of COVID-19 (HR 4.079, 95%CI, 1.086–15.322; *p* = 0.037) (9). Moreover, immunotherapy was early associated with a risk of worsening COVID-19 outcomes, as 4 out 6 lung cancer patients (66.7%) who were receiving immunotherapy died from COVID-19 (10). In Paris, the Gustave Roussy Cancer Center experience involved 137 cancer patients confirmed for COVID-19, of which 12 patients (10.1%) corresponded to thoracic malignancies (29). In univariate analysis, treatment with chemotherapy in the last 3 months significantly increased the risk of clinical worsening, with a HR 2.60 (1.32-5.13; *p* = 0.006) while neither targeted therapy nor immunotherapy had an impact in COVID-19 outcomes. On multivariate analysis, only performance status ECOG >1 remained statistically significant with a HR 3.9 (1.8-8.7; *p* = 0.008). In New York city, MSKCC experience analyzed 423 cancer patients with different tumor types and COVID-19, of which 35 (8%) corresponded to patients with lung cancer (30). In multivariate analysis, age ≥ 65 years and treatment with ICI within 90 days were predictors of hospitalization and severe disease, while chemotherapy within 30 days was not. However, an analysis performed from the same institution in New York focused specifically on lung cancer patients, found that treatment with anti-PD1 immune-checkpoint inhibitors was associated with smoking status, an independent predictor of severity of COVID-19 in lung cancer patients. After adjusting for smoking status, treatment with ICI did not show any significant association to COVID-19 severity, and IL-6 peak levels in hospitalized patients were similar irrespective to ICI therapy (31). In TERAVOLT registry, univariate analysis failed to demonstrate any influence on COVID-19 worsening outcome (hospitalization, prolonged hospitalization, or death) by type of treatment (none, TKI alone, chemo alone, ICI alone, chemo-immunotherapy, others) (25). More recently, in larger registries and prospective cohort studies in patients with cancer, neither cancer therapy nor type of anticancer therapy were associated with COVID-19 mortality (32, 33).

In the group of patients with lung cancer that were receiving active systemic cancer treatment during the outbreak period (*n* = 242), only male sex was associated with an increased risk of COVID-19 infection (OR 8.33). This sex predisposition to be affected by COVID-19 has been associated to a higher expression of angiotensin-converting enzyme 2 (ACE2) receptors in men compared to women (34). We tried to identify predictive factors of mortality in lung cancer patients with COVID-19. All predictive factors of mortality identified in our study were related to either tumor status (stage IV, progressive tumor, number of metastatic sites), clinical conditions (ECOG > 0, number of comorbidities) or COVID-19 severity status at diagnosis (low oxygen saturation, elevated fibrinogen and CRP). Number of metastatic sites can indirectly reflect the tumor burden. Previously, cardiovascular disease, diabetes, chronic respiratory disease and hypertension have been related to an increased risk of death by COVID-19 (12–15), common comorbidities found in patients with lung cancer as in our series. Moreover, inflammation markers have also been associated with worse COVID-19 outcomes (18). However, we were not able to find any independent factor that remained statistically significant in a multivariate model. We observed a high correlation among the different variables that were previously identified as statistically significant in the univariate analysis. This fact, together with the low number of deaths by COVID-19 in our series (*n* = 8), and that all patients with lung cancer who succumb to COVID-19 had stage IV disease, underpowered our study to find a robust predictive model of mortality. We have recent current evidence that general risk factors for COVID-19 are the most important predictors of mortality for patients with cancer. In the CCC19 global registry, with 928 patients with cancer and COVID-19 (10% with thoracic cancer, *n* = 91), an a mortality rate of 13%, independent factors of mortality identified were increased age (OR 1.84, 95% CI 1.53–2.21), male sex (1.63, 1.07–2.48), smoking status (former smoker vs. never smoked: 1.60, 1.03–2.47), number of comorbidities (two vs. none: 4.50, 1.33–15.28), ECOG >1 (3.89, 2.11–7.18), active cancer (5.20, 2.77–9.77), and azithromycin plus hydroxychloroquine treatment (2.93, 1.79–4.79) (33). In the UK, a prospective observational study involving 800 patients with cancer and COVID-19 (11% with thoracic malignancies, *n* = 90), 28% of patients died and risk of death was significantly associated with older age (OR 9.42 [95% CI 6.56–10.02]; *p* < 0.0001), male gender (1.67 [1.19–2.34]; *p* = 0.003), and the presence of comorbidities as hypertension (1.95 [1.36–2.80]; *p* < 0.001) and cardiovascular disease (2.32 [1.47–3.64]) (32). Specifically focused on lung cancer patients, both in the MSKCC experience and in TERAVOLT, baseline patients characteristics, age, smoking history, and comorbidities were the main mortality drivers from COVID-19 (24, 25). Our results support the key message that minimizing exposure is the most important preventive measure to protect patients with lung cancer.

Our study has also some limitations and include the relatively small sample size, that limited our power to draw formal conclusions in predictive factors of mortality as no more than 8 patients died in our series; not full representation of all different patients with lung cancer, with a bias toward a stage IV and an on active treatment population and limited surgical cases or on surveillance; highly impacted region by COVID-19 which may have influence patient outcomes, so our results have not necessarily to replicate in other regions of the world with wider access to full intensive supportive care for patients with lung cancer; short follow-up to identify both physical and psychological sequelae or detect some influence on cancer outcomes.

In conclusion, our series show that patients with lung cancer are extremely vulnerable to COVID-19. In a triage situation, ICU and IMV are not usually offered to patients with lung cancer and may account in the high mortality rate. Exposure to SARS-CoV-2 and clinical baseline conditions rather than treatment type are the main predictive factors of mortality. However, larger series are needed to confirm the latter finding, as the influence by treatment type on mortality cannot been ruled out due to the limited sample size of our study. Measures to minimize the risk of SARS-CoV-2 infection remain key to protect patients with lung cancer.

## Data Availability Statement

The raw data supporting the conclusions of this article will be made available by the authors, without undue reservation.

## Ethics Statement

The studies involving human participants were reviewed and approved by Comité de Ética de la Investigación con Medicamentos (CEIm) del Hospital General Universitario Gregorio Marañon (HGUGM). Written informed consent for participation was not required for this study in accordance with the national legislation and the institutional requirements. Written informed consent was not obtained from the individual(s) for the publication of any potentially identifiable images or data included in this article.

## Author Contributions

AC was responsible to conception and design, acquisition of data, analysis and interpretation of data, and drafting the article. The rest of the authors were responsible on acquisition of data, and/or analysis and interpretation of data, and revision of the draft article. All authors gave final approval of the final version submitted.

## Conflict of Interest

AC has received honorary/consulting fees from AstraZeneca, Boehringer-Ingelheim, Pfizer, Roche/Genentech, Eli Lilly and Company, Novartis, Merck Sharp & Dohme, and Bristol-Myers Squibb and travel/accommodations from Merck Sharp & Dohme, and Bristol-Myers Squibb, and Boehringer-Ingelheim. NG has received travel/accommodations from Merck Sharp & Dohme, Rovi, Sanofi-Aventis, Angelini. JS has 1st degree relatives working as employees in pharmaceutical industry (father, Pfizer; mother, Sanofi), has received travel/accommodations from Merck Sharp & Dohme, Angelini, Vifor Pharma, Rovi, Pfizer. TM has received honorary/consulting fees from AstraZeneca, Novartis, Roche and travel/accommodations from AstraZeneca, Novartis, Astellas. MG has received honorary/consulting fees and travel/accommodations from PharmaMar, and travel/accommodations from Lilly, MSD, and Roche. RÁ has received honorary/consulting fees from PharmaMar, Lilly, Novartis, Roche, Bristol-Myers Squibb, AstraZeneca, Boehringer-Ingelheim, and Takeda outside the submitted work. MM has received research grants from Roche, PUMA and Novartis, consulting/advisory fees from AstraZeneca, Amgen, Taiho Oncology, Roche/Genentech, Novartis, PharmaMar, Eli Lilly, PUMA, Taiho Oncology, Daiichi Sankyo and Pfizer and speakers' honoraria from AstraZeneca, Amgen, Roche/Genentech, Novartis, and Pfizer. The remaining authors declare that the research was conducted in the absence of any commercial or financial relationships that could be construed as a potential conflict of interest.

## Supplementary Material

Supplementary Figure 1 ROC curve with Youden index for neutrophil to lymphocyte ratio (NLR) in patients with lung cancer and COVID-19. In brackets, specificity and sensitivity, respectively. AUC, area under the curve.

Supplementary Table 1 Summary of the institutional protocol of treatment for COVID-19 in place at Hospital General Universitario Gregorio Marañon, Madrid (Spain) from March to May 2020.

Supplementary Table 2 Demographic characteristics of patients with lung cancer on active anti-tumor therapy by COVID-19 status attended at Hospital General Universitario Gregorio Marañon from February to May 2020 (*n* = 242).
