# Supplementary figures and images for: Outcomes of COVID-19 in Patients With Lung Cancer Treated in a Tertiary Hospital in Madrid
Source: Front Oncol. 2020 Sep 16;10:1777. doi: 10.3389/fonc.2020.01777 (PMC7525070; doi:10.3389/fonc.2020.01777)

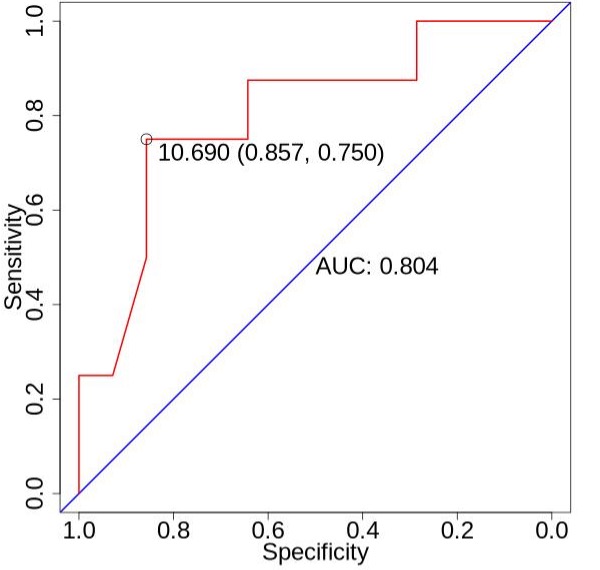

Supplement: Supplementary Figure 1 — ROC curve with Youden index for neutrophil to lymphocyte ratio (NLR) in patients with lung cancer and COVID-19. In brackets, specificity and sensitivity, respectively. AUC, area under the curve. [file Image_1.JPEG]
