# Supplementary material for: Outcomes of COVID-19 in Patients With Lung Cancer Treated in a Tertiary Hospital in Madrid
Source: Front Oncol. 2020 Sep 16;10:1777. doi: 10.3389/fonc.2020.01777 (PMC7525070; doi:10.3389/fonc.2020.01777)
Supplement: Supplementary Table 1 — Summary of the institutional protocol of treatment for COVID-19 in place at Hospital General Universitario Gregorio Marañon, Madrid (Spain) from March to May 2020. [file Table_1.DOCX]

|  | Protocol 15/03 | Protocol 26/03 | Protocol 03/04 |
| --- | --- | --- | --- |
| Mild pneumonia   - CURB 65: 1 - Sat > 90% | Hydroxychloroquine + Lopinavir/Ritonavir  or  Hydroxychloroquine + Azithromycin* | | |
| Severe pneumonia   - CURB-65 > 2 - Sat < 90% | Hydroxychloroquine + Lopinavir/Ritonavir  or  Hydroxychloroquine + Azithromycin*  +  Immunosuppressive/Immunomodulatory agents (According to criteria) | | |
| Remdesivir | Severe pneumonia with need for IMV | | |
|  | Immunosuppressive/Immunomodulatory agents | | |
| Interferón Beta-1b | Severe pneumonia | Withdrawn due to drug shortage | |
| Tocilizumab | Severe pneumonia with need for **IMV** or **NIV**  +  IL-6 > 40 pg/ml  or  D-dimer >1500 U/ml | Severe pneumonia with need for **IMV** and **ARDS**  Only one dose per patient  (drug shortage) | |
| Corticotherapy | Not recommended | Severe pneumonia > 60 years, comorbidity and increased APR  Glucocorticoids  Dexamethasone 20mg/d (5days) followed of 10mg/d (5days)  or  Metylprednisolone 1-2mg/kg/d  (3-5 days) | Severe pneumonia + ARDS (PaO2/FiO2 <300mmHg)  After 8 days from the onset of symptoms  Dexamethasone 40 mg/d (4days)  Or  Metylprednisolone 250 mg/d  (3 days) |
|  |  |  |  |
| Thromboprophylaxis | Not contemplated | | LMWH at prophylactic doses in hospitalized patients  (Maintain treatment 7/10 days after discharge from hospital)  LMWH at intermediate dose if  D-dimer > 3000 U/ml |
| Antibiotherapy | Suspected bacterial infection  (Increased procalcitonin, neutrophilia) | | |

**Supplementary Table 1.** Summary of the institutional protocol of treatment for COVID-19 in place at Hospital General Universitario Gregorio Marañon, Madrid (Spain) from March to May 2020.
