# Supplementary material for: Outcomes of COVID-19 in Patients With Lung Cancer Treated in a Tertiary Hospital in Madrid
Source: Front Oncol. 2020 Sep 16;10:1777. doi: 10.3389/fonc.2020.01777 (PMC7525070; doi:10.3389/fonc.2020.01777)
Supplement: Supplementary Table 2 — Demographic characteristics of patients with lung cancer on active anti-tumor therapy by COVID-19 status attended at Hospital General Universitario Gregorio Marañon from February to May 2020 (n = 242). [file Table_2.DOCX]

**Supplementary Table 2.** Demographic characteristics of patients with lung cancer on active anti-tumor therapy by COVID-19 status attended at Hospital General Universitario Gregorio Marañon from February to May 2020 (n=242)

|  |  | non-COVID-19 (n=231) | |  | COVID-19 (n=11) | |  | *P* value |
| --- | --- | --- | --- | --- | --- | --- | --- | --- |
|  |  |  |  |  |  |  |  |  |
|  |  |  |  |  |  |  |  |  |
| Age (years) | Median (range) |  | 68.2 (33-91) |  |  | 66.6 (49-86) |  | 0.8869 |
|  |  |  |  |  |  |  |  |  |
| Gender |  |  |  |  |  |  |  |  |
|  | Male | 127 | 55,0% |  | 10 | 90,9% |  | 0.0258 |
|  | Female | 104 | 45,0% |  | 1 | 9,1% |  |  |
|  |  |  |  |  |  |  |  |  |
| Smoking status |  |  |  |  |  |  |  |  |
|  | Current | 65 | 28,1% |  | 4 | 36,4% |  | 0.6829 |
|  | Former | 110 | 47,6% |  | 6 | 54,5% |  |  |
|  | Never | 43 | 18,6% |  | 1 | 9,1% |  |  |
|  | Unkown | 13 | 5,6% |  | 0 | 0,0% |  |  |
|  |  |  |  |  |  |  |  |  |
| ECOG PS |  |  |  |  |  |  |  |  |
|  | 0 | 63 | 27,3% |  | 1 | 9,1% |  | 0.8984 |
|  | 1 | 136 | 58,9% |  | 9 | 81,8% |  |  |
|  | 2 | 29 | 12,6% |  | 1 | 9,1% |  |  |
|  | >2 | 3 | 1,3% |  | 0 | 0,0% |  |  |
|  |  |  |  |  |  |  |  |  |
| Comorbidities |  |  |  |  |  |  |  |  |
|  | Yes | 173 | 74,9% |  | 9 | 81,8% |  | 0.6032 |
|  | No | 58 | 25,1% |  | 2 | 18,2% |  |  |
|  |  |  |  |  |  |  |  |  |
| Pathology |  |  |  |  |  |  |  |  |
|  | Adenocarcinoma | 155 | 67,1% |  | 8 | 72,7% |  | 0.6137 |
|  | SqCC | 33 | 14,3% |  | 2 | 18,2% |  |  |
|  | SCLC | 30 | 13,0% |  | 0 | 0,0% |  |  |
|  | Others | 13 | 5,6% |  | 1 | 9,1% |  |  |
|  |  |  |  |  |  |  |  |  |
| Stage |  |  |  |  |  |  |  |  |
|  | I-II | 6 | 2,6% |  | 0 | 0,0% |  | 0.8542 |
|  | III | 45 | 19,5% |  | 2 | 18,2% |  |  |
|  | IV | 180 | 77,9% |  | 9 | 81,8% |  |  |
|  |  |  |  |  |  |  |  |  |
| Treatment |  |  |  |  |  |  |  |  |
|  | Chemotherapy | 112 | 48,5% |  | 5 | 45,5% |  | 0.2474 |
|  | Immunotherapy | 51 | 22,1% |  | 5 | 45,5% |  |  |
|  | Targeted therapy | 51 | 22,1% |  | 1 | 9,1% |  |  |
|  | Chemo + immunotherapy | 17 | 7,4% |  | 0 | 0,0% |  |  |
|  |  |  |  |  |  |  |  |  |
|  |  |  |  |  |  |  |  |  |
| PS, performance status; SqCC, squamous cell carcinoma; SCLC, small-cell lung cancer. | | | | | | |  |  |
